# Not only baseline but cumulative exposure of remnant cholesterol predicts the development of nonalcoholic fatty liver disease: a cohort study

**DOI:** 10.1265/ehpm.23-00289

**Published:** 2024-02-06

**Authors:** Lei Liu, Changfa Wang, Zhongyang Hu, Shuwen Deng, Saiqi Yang, Xiaoling Zhu, Yuling Deng, Yaqin Wang

**Affiliations:** 1 Health Management Center, The Third Xiangya Hospital, Central South University, No. 138 Tongzipo Road, Yuelu District, Changsha, Hunan, China, 410013; 2 General Surgery Department, The Third Xiangya Hospital, Central South University, No. 138 Tongzipo Road, Yuelu District, Changsha, Hunan, China, 410013; 3 Department of Neurology, The Third Xiangya Hospital, Central South University, No. 138 Tongzipo Road, Yuelu District, Changsha, Hunan, China, 410013

**Keywords:** Remnant cholesterol, Nonalcoholic fatty liver disease, Cumulative exposure, Cohort study, Epidemiology

## Abstract

**Background and aim:**

Remnant cholesterol (remnant-C) mediates the progression of major adverse cardiovascular events. It is unclear whether remnant-C, and particularly cumulative exposure to remnant-C, is associated with nonalcoholic fatty liver disease (NAFLD). This study aimed to explore whether remnant-C, not only baseline but cumulative exposure, can be used to independently evaluate the risk of NAFLD.

**Methods:**

This study included 1 cohort totaling 21,958 subjects without NAFLD at baseline who underwent at least 2 repeated health checkups and 1 sub-cohort totaling 2,649 subjects restricted to those individuals with at least 4 examinations and no history of NAFLD until Exam 3. Cumulative remnant-C was calculated as a timeweighted model for each examination multiplied by the time between the 2 examinations divided the whole duration. Cox regression models were performed to estimate the association between baseline and cumulative exposure to remnant-C and incident NAFLD.

**Results:**

After multivariable adjustment, compared with the quintile 1 of baseline remnant-C, individuals with higher quintiles demonstrated significantly higher risks for NAFLD (hazard ratio [HR] 1.48, 95%CI 1.31–1.67 for quintile 2; HR 2.07, 95%CI 1.85–2.33 for quintile 3; HR 2.55, 95%CI 2.27–2.88 for quintile 4). Similarly, high cumulative remnant-C quintiles were significantly associated with higher risks for NAFLD (HR 3.43, 95%CI 1.95–6.05 for quintile 2; HR 4.25, 95%CI 2.44–7.40 for quintile 3; HR 6.29, 95%CI 3.59–10.99 for quintile 4), compared with the quintile 1.

**Conclusion:**

Elevated levels of baseline and cumulative remnant-C were independently associated with incident NAFLD. Monitoring immediate levels and longitudinal trends of remnant-C may need to be emphasized in adults as part of NAFLD prevention strategy.

**Supplementary information:**

The online version contains supplementary material available at https://doi.org/10.1265/ehpm.23-00289.

## 1. Introduction

Nonalcoholic fatty liver disease (NAFLD), characterized by excessive intrahepatic lipid accumulation, is the most prevalent chronic liver disease in the world [1, 2]. It is the hepatic manifestation of metabolic syndrome and progressed from simple steatosis to nonalcoholic steatohepatitis, cirrhosis and hepatocellular carcinoma [3]. NAFLD not only brings a serious burden of liver-related diseases but also affects multiple organ systems in the whole body outside the liver, including the cardiocerebrovascular system, musculoskeletal system, et al [4]. Evolving data from meta-analyses and cohort studies support the notion that the most common cause of death in the NAFLD population is cardiovascular disease (CVD) [5–7]. Nevertheless, there is no specific drugs for the treatment of NAFLD to date, novel biomarkers to identify individuals at high risk of incident and development of NAFLD are urgently required [8].

Because intrahepatic lipid accumulation results from lipid metabolism abnormalities, it takes for granted that dyslipidemia can cause NAFLD [9]. Lowering low-density lipoprotein cholesterol (LDL-C) level with statins is deemed the primary pharmacological therapy and treatment goals for CVD prevention, however, patients with large decreases in LDL-C levels still have considerable CVD risks, that is, residual risks [10]. In recent years, the goal of reclassification of high-risk CVD populations has shifted from simply focusing on LDL-C to nontraditional lipids, such as remnant cholesterol (remnant-C) [11, 12]. Remnant-C is the cholesterol content of triglyceride-rich lipoproteins (TRLs) that consists of very-low and intermediate density lipoproteins in the fasting state and chylomicron remnants in the nonfasting state [13]. Recently, studies have explored the relationship between remnant-C and NAFLD and found that remnant-C was associated with NAFLD independent of other risk factors [1, 9, 14–18]. However, most of these previous studies were cross-sectional which cannot reflect the causal relationship. Furthermore, these studies relied on the baseline single-point remnant-C measurement, and thus could not investigate the associations of changes in remnant-C levels over time with future NAFLD development.

In our prospective cohort study, we aim to investigate the association between baseline and cumulative remnant-C exposure to the development of NAFLD in the Chinese health check-up population, in an attempt to expand our understanding of the remnant-C as a potential risk factor of NAFLD. To our knowledge, our study is the first analysis specifically led to evaluate the effect of cumulative remnant-C exposure to the development of NAFLD.

## 2. Materials and methods

### 2.1 Study design and study population

**Fig. 1:**
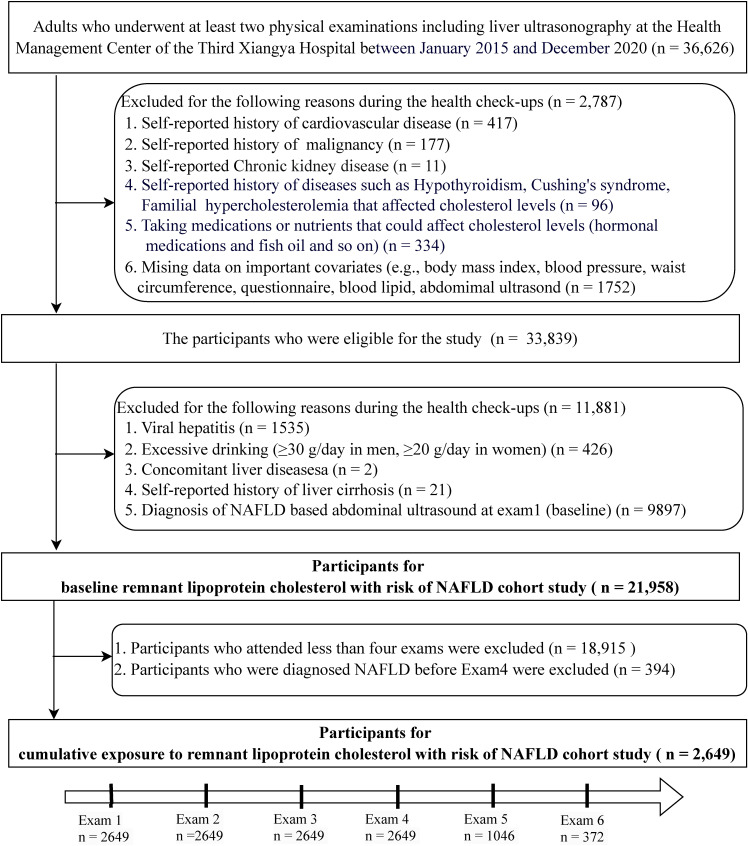
Study flowchart.

This cohort study was derived from an ongoing longitudinal study in the Third Xiangya Hospital of Central South University (Changsha city, China), the largest medical institution in Central China, of which a detailed description has been published [19, 20]. Participants were derived from the employee and retiree populations. From January 2015 to December 2020, a total of 36,626 adults were identified as having had at least 2 electronic records of health checkups including liver ultrasonography. We further excluded individuals with a history of cardiovascular disease, chronic kidney disease, cancer, hypothyroidism, Cushing’s syndrome, familial hypercholesterolemia, taking medications or nutrients that could affect cholesterol levels (hormonal medications, fish oil and so on) and incomplete data (n = 2,787), and those with diagnosed NAFLD at baseline and concomitant liver diseases (n = 11,881). Thus, a total of 21,958 participants were included in the baseline remnant-C with risk of NAFLD cohort study. Of these participants, 2,649 individuals with at least four examinations and who did not have a diagnosis of NAFLD prior to Exam 4, were included in the cumulative exposure to remnant-C with risk of NAFLD cohort study (Fig. 1).

All participants provided written informed consent and the Ethics Committee at the Third Xiangya Hospital of Central South University (No. 2013BAI04B01) approved the study.

### 2.2 Clinical characteristics

Data on demographics, lifestyle, and self-reported disease history were obtained using the National Unified Physical Examination Questionnaire via a website (https://new.selfhealth.com.cn/#/login). Current smokers were defined as smoking more than one cigarette per day (on average) over a period longer than 6 months. Regular physical activity was defined as the aerobic exercise performed more than 3 times per week with at least thirty minutes each time. Current drinkers were defined consumption of beer, wine (including Chinese wine) and/or liquor at least two days per week over a period exceeding 12 months.

Anthropometric measurements conducted by trained physicians or nurses. Weight and height were measured with the participants wearing light clothing and no shoes. Body mass index (BMI) was calculated as weight in kilograms divided by height in meters squared. Waist circumference (WC) was measured using nonelastic tape at a level midway between the bottom edge of the last rib and iliac crest with the individuals standing up and the abdomen relaxed. Systolic blood pressure (SBP) and diastolic blood pressure (DBP) of the right upper arm was measured using an automatic sphygmomanometer (OMRON 9020) with participants resting for at least 5 min prior to taking measurements.

Blood samples taken in the morning after an overnight fast (8–12 h) were analyzed at the clinical laboratory of Third Xiangya Hospital with an automatic analyzer (Hitachi 7600-110; Hitachi, Tokyo, Japan). Blood chemistry was conducted including fasting plasma glucose, alanine aminotransferase (ALT), creatinine, total cholesterol (TC), high density lipoprotein cholesterol (HDL-C) and low-density lipoprotein cholesterol levels (LDL-C) and triglycerides (TG). Lipid measurements were determined by enzymatic methods. Low–density lipoprotein cholesterol (LDL–C) was measured directly [21]. The estimated glomerular filtration rate (eGFR) was calculated by the Modification of Diet in Renal Disease formula for Chinese subjects [22]. The diagnostic criteria for hypertension, diabetes and dyslipidemia were provided in Supplementary Table S1.

### 2.3 Definition of baseline and cumulative exposure to remnant-C

Remnant-C was calculated as TC level minus the LDL-C level minus the HDL-C level. The baseline remnant-C was the level of remnant-C at exam1. The cumulative exposure to remnant-C was assessed by a time weighted model calculated using all available remnant-C measurements from baseline to the end of this study or before any incident NAFLD as previously reported [23]. This equation was (Remnant-C_Exam1_ × time_1–2_ + Remnant-C_Exam2_ × time_2–3_ + Remnant-C_Exam3_ × time_3–4_ + Remnant-C_Exam4_ × time_4–5_ + Remnant-C_Exam5_ × time_5–6_)/the follow-up duration (time_1–2_ + time_2–3_ + time_3–4_ + time_4–5_ + time_5–6_), where time_n–n+1_ indicate the interval time between the two consecutive exams from Exam_n_ to Exam_n+1_ in years.

### 2.4 Assessment of new-onset NAFLD

In line with the Chinese Liver Disease Association [24], participants were diagnosed with NAFLD as the presence of hepatic steatosis without excessive alcohol consumption (≥30 g/d in men and ≥20 g/d in women) or concomitant liver disease (viral hepatitis, alcoholic liver disease, toxic liver disease, autoimmune hepatitis, etc). Hepatic steatosis was assessed using abdominal ultrasonography using a 3.5-MHz probe (Logiq 9, GE Medical System, Milwaukee, WI, USA) by experienced radiologists who were blinded to the study design. Positive liver fat was identified as standard criteria of increased liver echogenicity, as presence two of the following three criteria: liver-to-kidney or spleen contrast, vascular blurring and deep beam attenuation based on the Asia–Pacific Working Party recommendations [25, 26].

### 2.5 Statistical analyses

Continuous variables of baseline characteristics were described as means ± SD or median (first quartile, third quartile) and were analyzed using a linear trend test, a generalized linear model across the quartiles of baseline remnant-C. Categorical variables were presented as numbers (percentages) and were compared using the Wilcoxon test or the χ^2^ test.

Follow-up time was calculated as the interval from baseline (the date of Exam1) to the first occurrence of NAFLD or the last examination if incident NAFLD had not been identified, whichever came first. The incidence of NAFLD outcomes was calculated by dividing the number of events by the total person-years during the whole follow-up time. Multivariate Cox proportional hazards regression analyses were used to explore the risk of incident NAFLD associated with baseline and cumulative remnant-C. Proportional hazards (PH) were examined using Schoenfeld residuals analysis and Log-log plot. *P* values for linear trend across increasing quartiles were conducted by entering median value in each quartile as a continuous variable in Cox regression models.

Covariables were selected based on possible risks factors with NAFLD or associated with remnant-C in univariate analysis with a value of p < 0.10. In addition, BMI was selected instead of WC and SBP instead of DBP due to multicollinearity. We finally fitted 3 models: Model 1 was adjusted by age and sex; Model 2 was additionally adjusted by education level, current drinking, current smoking, physical activity, BMI, SBP, fasting glucose, triglycerides, eGFR, and ALT at exam1; Model 3 was further adjusted by antidiabetic, lipid-lowering, or antihypertensive medications usage at exam1 for baseline remnant-C, and medications usage before last exam for cumulative remnant-C exposure.

To model the dose-response curves of baseline remnant-C, as continuous change, with incident NAFLD, we used a restricted cubic spline with 3 knots at the 10th, 50th, and 90th percentiles, adjusting for the same variables as in the Cox regression analyses. To explore whether our findings varied by specific sub-cohorts, we evaluated the relationship between baseline remnant-C and incident NAFLD across different obesity subphenotypes and performed subgroup analyses stratified by sex (male or female), age (<40 or ≥40 years), hypertension (yes or no), diabetes (yes or no) and dyslipidemia (yes or no).

To confirm the robustness of our estimates, we performed several sensitivity analyses by repeating the Cox proportional hazards models: (i) excluding individuals on statin therapy (n = 21,757) and (ii) using the definition of metabolic dysfunction-associated fatty liver disease (MAFLD) instead of NAFLD (n = 23,766) for baseline remnant-C with risk of NAFLD analysis; (iii) the outcome using the incident NAFLD at Exam4 instead the last exam for cumulative remnant-C with incident NAFLD analysis (n = 2,649); (iv) including participants with a minimum of three visits and who did not have a diagnosis of NAFLD prior to Exam 3 (n = 7,718). The definitions of obesity sub-phenotypes and MAFLD were presented in Supplementary Table S1.

All analyses were performed using R version 4.2.3 (R Foundation for Statistical Computing, Vienna, Austria). The “survival” package in R was used to conduct Cox proportional hazard regression analysis. The “rms” and “ggplot2” packages were used to perform restricted cubic spline analysis. A two-sided *P* value <0.05 were regarded as statistically significant.

## 3. Results

### 3.1 Baseline characteristics of the study population

A total of 21,958 individuals, with a mean ± SD age of 39.4 ± 12.1 years, was included in the study. Baseline characteristics are summarized in Table 1. In general, participants in the higher remnant-C quartiles were more likely to be male and older; to be less educated; to be a current smoker and current alcohol drinker; to be less physically active; to be having worse cardiovascular metabolic traits than participants in the lower remnant-C quartiles.

**Table 1:** Baseline characteristics according to quartiles of baseline remnant-C (n = 21,958).

**Characteristics**	**Overall**	**Quartile 1**	**Quartile 2**	**Quartile 3**	**Quartile 4**	***P* for trend**

		**<0.35 mmol/L**	**0.35–0.49 mmol/L**	**0.49–0.72 mmol/L**	**≥0.72 mmol/L**	
**Prevalence, n (%)**	21958 (100.0)	5398 (24.6)	5570 (25.4)	5524 (25.2)	5466 (24.9)	
**Demographic factors**						
Age, years	39.4 ± 12.1	34.6 ± 10.2	38.3 ± 11.7	41.0 ± 12.3	43.4 ± 12.1	<0.001
Female, n (%)	12804 (58.3)	4297 (79.6)	3701 (66.5)	2878 (52.1)	1928 (35.3)	<0.001
University degree, n (%)	15933 (72.6)	4177 (77.4)	4153 (74.6)	3903 (70.7)	3700 (67.7)	<0.001
**Lifestyle status**						
Current smoker, n (%)	4064 (18.5)	360 (6.7)	738 (13.3)	1129 (20.4)	1837 (33.6)	<0.001
Current drinker, n (%)	5167 (23.5)	734 (13.6)	1062 (19.1)	1345 (24.4)	2026 (37.1)	<0.001
Regular exercise, n (%)	6285 (28.6)	1619 (30.0)	1600 (28.7)	1568 (28.4)	1498 (27.4)	0.028
**Cardiovascular metabolic factors**						
BMI, kg/m^2^	22.5 ± 2.8	21.12 ± 2.3	22.0 ± 2.5	22.8 ± 2.6	24.2 ± 2.9	<0.001
WC, cm	76.5 ± 8.7	71.4 ± 6.9	74.5 ± 7.6	77.7 ± 7.8	82.3 ± 8.4	<0.001
Systolic blood pressure, mm Hg	118.1 ± 15.2	112.9 ± 12.5	115.6 ± 13.9	119.2 ± 14.9	124.8 ± 16.4	<0.001
Diastolic blood pressure, mm Hg	72.4 ± 10.6	68.6 ± 9.0	70.7 ± 9.6	73.0 ± 10.1	77.4 ± 11.7	<0.001
Hypertension, n (%)	2559 (11.7)	204 (3.8)	412 (7.4)	670 (12.1)	1273 (23.3)	<0.001
Anti-hypertensive medication, n (%)	1106 (5.0)	75 (1.4)	164 (2.9)	294 (5.3)	573 (10.5)	<0.001
Fasting glucose, mmol/L	5.2 ± 0.9	5.0 ± 0.6	5.1 ± 0.6	5.2 ± 0.8	5.5 ± 1.3	<0.001
Diabetes mellitus, n (%)	767 (3.5)	64 (1.2)	102 (1.8)	208 (3.8)	393 (7.20)	<0.001
Anti-diabetes medication, n (%)	488 (2.2)	55 (1.0)	77 (1.4)	138 (2.5)	218 (4.0)	<0.001
TC, mmol/L	4.8 ± 0.9	4.4 ± 0.7	4.7 ± 0.8	5.0 ± 0.9	5.3 ± 1.0	<0.001
Triglycerides, mmol/L	1.1 (0.8, 1.5)	0.6 (0.5, 0.7)	0.9 (0.8, 1.0)	1.3 (1.2, 1.4)	2.0 (1.7, 2.7)	<0.001
HDL-cholesterol, mmol/L	1.5 ± 0.3	1.6 ± 0.3	1.5 ± 0.3	1.4 ± 0.3	1.3 ± 0.3	<0.001
LDL-cholesterol, mmol/L	2.78 ± 0.8	2.5 ± 0.6	2.8 ± 0.7	2.9 ± 0.8	3.0 ± 0.9	<0.001
Dyslipidemia, n (%)	4328 (19.7)	187 (3.5)	452 (8.1)	829 (15.0)	2860 (52.3)	<0.001
Anti-dyslipidemia medication, n (%)	201 (0.9)	1 (0.0)	9 (0.2)	24 (0.4)	167 (3.1)	<0.001
**Emerging related factors**						
ALT, U/L	17.0 (13.0, 25.0)	14.0 (11.0, 19.0)	16.0 (12.0, 22.0)	18.0 (14.0, 25.0)	24.0 (17.0, 33.0)	<0.001
eGFR, mL/min/1.73 m^2^	112.9 (98.1, 130.1)	121.3 (107.1, 138.4)	115.1 (100.3, 132.1)	109.3 (95.3, 125.7)	105.9 (92.2, 122.0)	<0.001

Values are frequency (percentage), mean ± SD, or median (first quartile, third quartile).

Abbreviations: BMI, Body mass index; WC, waist circumference; TC, total cholesterol; HDL, high-density lipoprotein; LDL, low-density lipoprotein; ALT, alanine aminotransferase; eGFR, estimated glomerular filtration rate.

### 3.2 Baseline remnant-C and risk of incident NAFLD

After a median of 2.0 years (interquartile range, 1.3–3.0 years) of follow-up, 5,174 (23.6%) NAFLD were ascertained (incidence rate, 99.9 cases per 1000 person-years). Among the participants in the quintile 1, quintile 2, quintile 3, and quintile 4 of baseline remnant-C, the incidence of NAFLD gradually increased (30.96, 60.45, 107.95, and 202.66 per 1000 person-years, respectively) (Table 2).

**Table 2:** Association of baseline remnant-C with risk of NAFLD in Cox proportional hazard models (n = 21,958).

	**Baseline Remnant-C quartiles**				*P* for trend*

	**Quartile 1** **<0.35 mmol/L**	**Quartile 2** **0.35–0.49 mmol/L**	**Quartile 3** **0.49–0.72 mmol/L**	**Quartile 4** **≥0.72 mmol/L**	
**Total, n**	5398	5570	5524	5466	
Case number, n (%)	393 (7.28)	809 (14.52)	1408 (25.49)	2564 (49.91)	
Incidence rate per 1,000	30.96	60.45	107.95	202.66	
Model 1	1.00 (Reference)	1.62 (1.44–1.83)	2.52 (2.25–2.83)	4.08 (3.65–4.56)	<0.001
Model 2	1.00 (Reference)	1.49 (1.32–1.68)	2.08 (1.86–2.34)	2.54 (2.26–2.87)	<0.001
Model 3	1.00 (Reference)	1.48 (1.31–1.67)	2.07 (1.85–2.33)	2.55 (2.27–2.88)	<0.001

Model 1 was adjusted for age (years), sex.

Model 2 was adjusted for model 1 plus education level, current drinking, current smoking, physical activity, BMI, systolic blood pressure, fasting glucose, triglycerides, estimated GFR, and alanine transaminase at exam1.

Model 3 was adjusted for model 2 plus antidiabetic, lipid-lowering, or antihypertensive medications usage at exam1.

*Test for trend based on variable containing median value for each quarter.

**Fig. 2:**
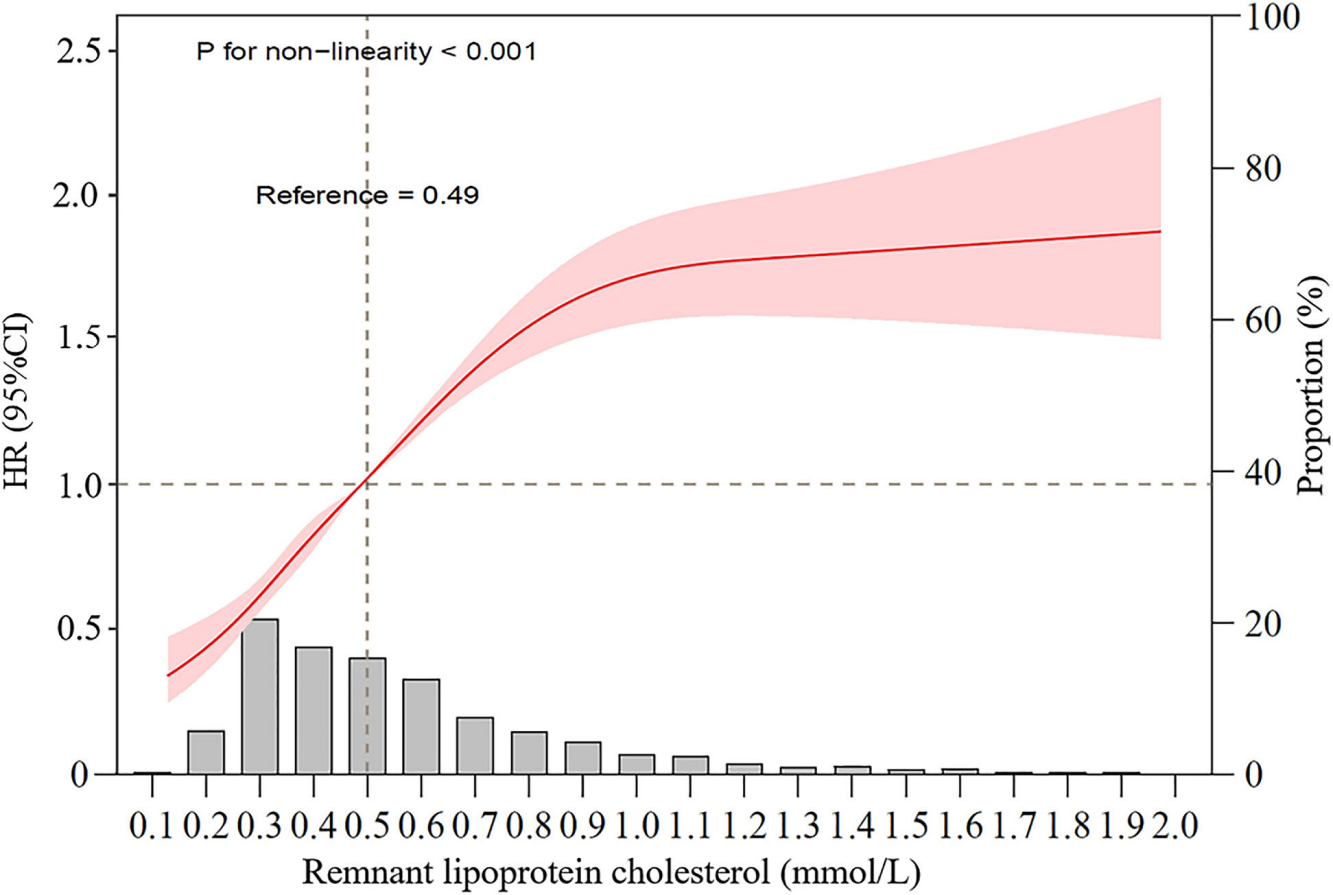
Restricted cubic spline graph of remnant-C and risk of incident NAFLD. Data were fitted using a Cox regression model of the restricted cubic spline with 3 knots at 10th, 50th, and 90th percentiles of baseline remnant-C level. HR hazard ratio, CI confidence interval. The reference point was the median of the remnant lipoprotein cholesterol in the 21,958 participants. The solid line represented point estimation on the association of remnant lipoprotein cholesterol with the development of NAFLD, and the shading indicates 95% CI estimations. The histogram represents the frequency distribution of remnant-C among study participants. Covariates in the model included age, sex, education level, current drinking, current smoking, physical activity, BMI, systolic blood pressure, fasting glucose, triglycerides, estimated GFR, and alanine transaminase, antidiabetic, lipid-lowering, or antihypertensive medications usage at exam1.

Table 2 shows the adjusted hazard ratios (aHRs) of incident NAFLD associated with quintiles of baseline remnant-C level. In the fully adjusted model, compared with participants in quintile 1, the aHRs were 1.48 (95% CI, 1.31–1.67) for quintile 2, 2.07 (95% CI, 1.85–2.33) for quintile 3 and 2.55 (95% CI, 2.27–2.88) for quintile 4 (*P* for trend < 0.001). In the subgroup analyses, the effect was consistent across sex, age, hypertension, diabetes and dyslipidemia, but no statistical interactions were found (Supplementary Fig. S1). Likewise, a cubic spline curve showing the association between baseline remnant-C and incident NAFLD was nonlinear, which presented the trend of increasing risk of NAFLD development with increasing level of remnant-C (Fig. 2).

**Fig. 3:**
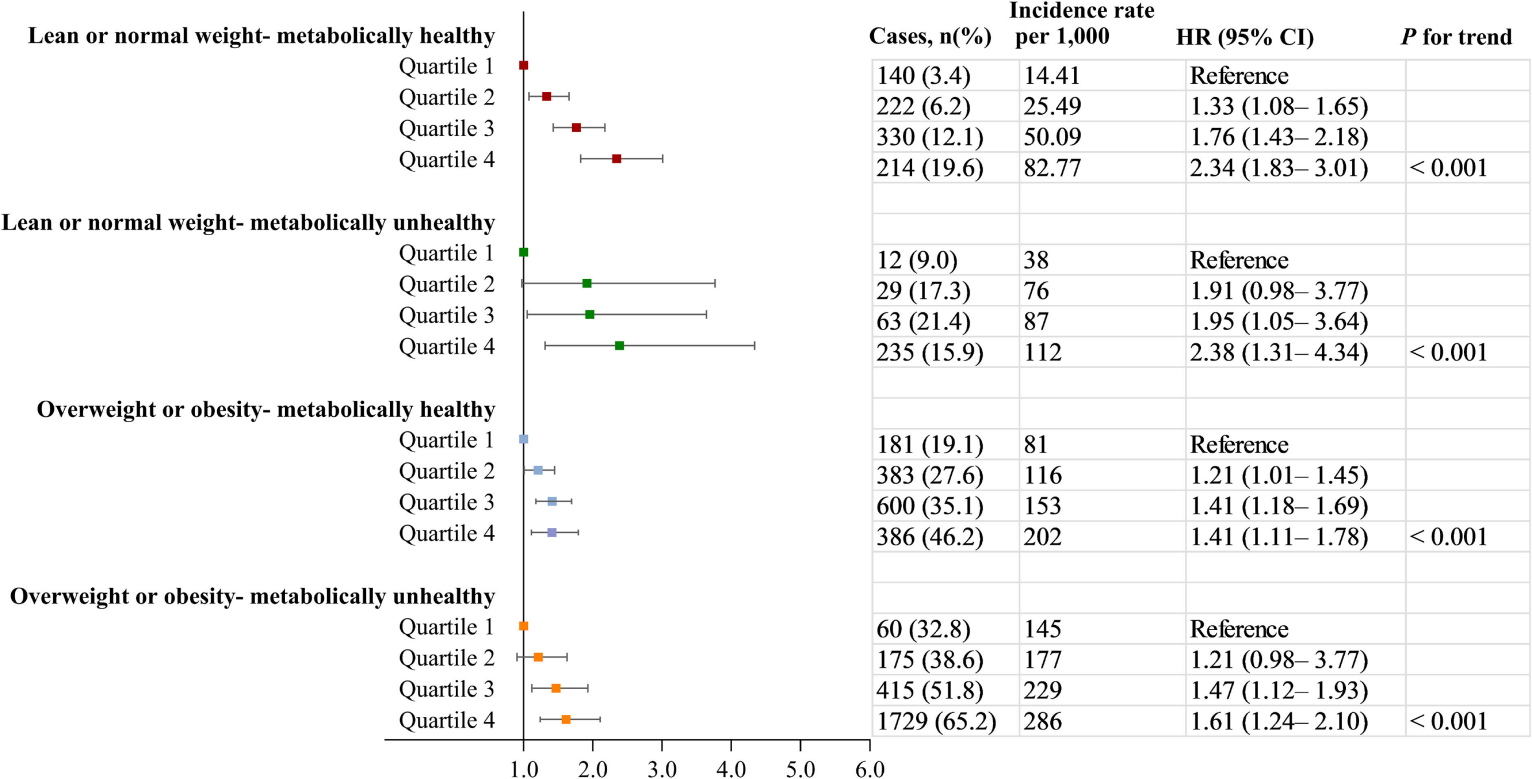
Associations between remnant-C and the incidence of NAFLD in people with different obesity sub-phenotypes. Data were adjusted for age, sex, education level, current drinking, current smoking, physical activity, BMI, systolic blood pressure, fasting glucose, triglycerides, estimated GFR, and alanine transaminase, antidiabetic, lipid-lowering, or antihypertensive medications usage at exam1. CI, confidence interval; HR hazard ratio; NAFLD, non-alcoholic fatty liver disease.

Adopting the clinical definitions of obesity status and metabolic health, we further explored the relationship between remnant-C and incident NAFLD across different obesity sub-phenotypes. After multivariable adjustment, remnant-C was still significantly associated with a higher risk of incident NAFLD in these subtypes. Observational aHRs for 1 quintile versus 4 quintile of remnant-C were 2.34 (95% CI: 1.83–3.01) for lean or normal weight - metabolically healthy subtype, 2.38 (95% CI: 1.31–4.34) for lean or normal weight - metabolically unhealthy subtype, 1.41 (95% CI: 1.11–1.78) for overweight or obesity - metabolically healthy subtype, 1.61 (95% CI: 1.24–2.10) for overweight or obesity - metabolically unhealthy subtype (Fig. 3).

### 3.3 Cumulative remnant-C exposure and risk of incident NAFLD

After a median of 4.0 years (interquartile range, 3.1–5.0 years) of follow-up, 360 (13.6%) NAFLD were ascertained (incidence rate, 33.74 cases per 1000 person-years). Among the participants in the quintile 1, quintile 2, quintile 3, and quintile 4 of cumulative remnant-C, the incidence of NAFLD gradually increased (5.95, 24.19, 34.35, and 71.42 per 1000 person-years, respectively) (Table 3).

**Table 3:** Association of cumulative exposure to remnant-C with NAFLD in Cox proportional hazard models (n = 2,649).

	**Cumulative Remnant-C quartiles**				***P* for trend**

	**Quartile 1** **<0.36 mmol/L**	**Quartile 2** **0.36–0.47 mmol/L**	**Quartile 3** **0.47–0.64 mmol/L**	**Quartile 4** **≥0.64 mmol/L**	
**Total, n**	662	659	666	662	
Case number, n (%)	16 (2.42)	65 (9.86)	92 (13.81)	187 (28.25)	
Incidence rate per 1,000	5.95	24.19	34.35	71.42	
Model 1	1.00 (Reference)	3.44 (1.98–5.95)	4.63 (2.71–7.91)	8.09 (4.79–13.66)	<0.001
Model 2	1.00 (Reference)	3.40 (1.94–5.99)	4.24 (2.43–7.37)	6.41 (3.67–11.18)	<0.001
Model 3	1.00 (Reference)	3.43 (1.95–6.05)	4.25 (2.44–7.40)	6.29 (3.59–10.99)	<0.001

Model 1 was adjusted for age (years), sex.

Model 2 was adjusted for model 1 plus education level, current drinking, current smoking, physical activity, BMI, systolic blood pressure, fasting glucose, triglycerides, estimated GFR, and alanine transaminase at exam1.

Model 3 was adjusted for model 2 plus antidiabetic, lipid-lowering, or antihypertensive medications usage before last exam.

*Test for trend based on variable containing median value for each quarter.

Table 3 shows the aHRs of incident NAFLD associated with quintiles of cumulative remnant-C level. After multivariable adjustment, the aHRs of incident NAFLD increased steadily as the quintiles of cumulative remnant-C increased. In the fully adjusted model, compared with participants in quintile 1, the aHRs were 3.43 (95% CI, 1.95–6.05) for quintile 2, 4.25 (95% CI, 2.44–7.40) for quintile 3 and 6.29 (95% CI, 3.59–10.99) for quintile 4 (*P* for trend < 0.001). Furthermore, the association of remnant-C level with the development of NAFLD was attenuated after using the baseline remnant-C exposure substitute the cumulative remnant-C exposure (Supplementary Table S2).

### 3.4 Sensitivity analysis

A sensitivity analysis with restriction of our sample to participants without taking lipid-lowering medications showed similar associations between baseline remnant-C and incident NAFLD (Supplementary Table S3). Similarly, the use of an alternative definition of MAFLD (based on the international expert consensus statement of 2020 led to a slight enhance of the magnitude of the associations [27]. However, this did not generally affect the statistical significance of these associations (Supplementary Table S4). The association of cumulative remnant-C with NAFLD risk was also not materially altered, in which incident NAFLD events that occurred until Exam4 (Supplementary Table S5). Additionally, similar results were found in the Cox regression model among participants with a minimum of three visits (Supplementary Table S6).

## 4. Discussion

### 4.1 Main findings and highlights

In this prospective cohort based on large health check-up population, not only baseline but cumulative exposure of remnant-C levels was positively associated with NAFLD after adjusting for variable confounders and robust sensitivity analyses, which confirmed the previous evidence and expand the understanding on the causal role of remnant-C in NAFLD. Moreover, the relationship between remnant-C and NAFLD is likely to be underestimated when a single exposure measure at baseline is used as opposed to a cumulative measure. These findings thus provide the first evidence that mid-term exposure to high level of remnant-C is not only strongly associated with future onset of NAFLD but that it is likely to be a more accurate index of the true magnitude of risk as compared with a single measure of remnant-C done several years before the incidence of NAFLD.

### 4.2 Comparisons with other studies and what does the current work add to the existing knowledge

Dyslipidemia is a recognized pathogenic factor of NAFLD and has been confirmed in several epidemiological and genetic studies [28, 29]. Patients with large decreases in LDL-C levels still have considerable CVD risks, which makes us have to pay more attention to the existence of residual risks. Considerable epidemiological and genetic studies confirmed that remnant-C increased the risk of major adverse cardiovascular event (MACEs) [30]. However, the causal relationship between residual cholesterol and NAFLD is still unclear. Recently, increasing cross-sectional studies have explored the relationship in both adults and adolescents and found that serum remnant-C was positively correlated with the risks of prevalent NAFLD independent of traditional risk factors [1, 14, 15, 31]. In 2023, Cheng et al. conducted a longitudinal retrospective cohort study including included 6,634 Chinese participants with an average follow-up time of 43.34 months and found that levels of triglycerides and remnant-C, but not TC or LDL-C, were associated with NAFLD outcomes independent of other risk factors [9]. Similarly, Miao et al. and Huang et al. conducted the similar conclusion in a longitudinal cohort consisted of 16,173 non-obese individuals within the 5-year follow-up period and in a prospective cohort which included 9,184 adults with a total of 31,662 person-years of follow-up, respectively [16, 17]. However, all of these studies focused on the baseline single-point rather than the dynamic cumulative exposure of remnant-C contributing to NAFLD. Our findings extend previous work by demonstrating for the first time that high level of remnant-C is associated with a substantially higher risk of incident NAFLD compared with those with low level of remnant-C when measured either at baseline or cumulatively. Furthermore, we analyzed the dose-response effect and found that the association between baseline remnant-C and incident NAFLD was nonlinear, which was consistent with that reported by Chen et al [18].

In the subgroup analyses of our study, there were no statistical interactions for sex, age, and history of hypertension, diabetes and dyslipidemia, which was similar to that reported by Huang et al. and Miao et al. [16, 17], but was different from the results published by Zou et al. and Chen et al [15, 18]. Zou et al. found that there were significant differences in NAFLD risk among remnant-C patients stratified by age, sex and BMI [15]. And Chen et al. identified age (P for interaction = 0.0309) and waist circumference (P for interaction = 0.0071) as interaction factors in subgroup analysis [18]. The differences in subgroup interaction analysis among aforementioned studies may be due to diverse study subtypes, sample sizes, age proportions, sex ratios and follow-up times. Furthermore, we performed stratified analyses to assess the effect of baseline remnant-C on incident NAFLD across various obesity sub-phenotypes, and we found that the relationship between remnant-C and NAFLD was consistent across different body shapes and metabolic status. These findings revealed that remnant-C serves as a promising marker or potential treatment target of NAFLD independent of traditional risk factors, which is important for perfect health care resource allocation and prevention and management of NAFLD, in turn to attenuate the society medical burden.

### 4.3 Mechanism of the link between remnant-C and NAFLD

The underlying mechanisms by which remnant-C was positively related to the risk of NAFLD development remains unknown. Remnant-C is the cholesterol content of TRLs which refer to non-high-density lipoprotein and non-low-density lipoprotein, both of which contain mostly cholesterol rather than triglycerides. The cholesterol content was hypothesized to be the major atherosclerotic component of TRLs [32]. First, the triglyceride content of TRLs was hydrolyzed by lipoprotein lipase, reduced activity of lipoprotein lipase resulted in inadequate clearance of TRLs [33, 34]. And then the activation of lipoprotein lipase has been shown to be effective in reducing the severity of hepatic steatosis [35]. Therefore, it is hypothesized that remnant-C contributing NAFLD due to delayed triglyceride metabolism. Second, given that low-grade systematic inflammation plays a pivotal role in the pathogenesis of NAFLD, previous studies reported that remnant-C was positively linked to low-grade systematic inflammation indicated by C-reactive protein and tumor necrosis factor α, we speculated that low-grade systematic inflammation may partly account for the association of remnant-C with NAFLD [36–38]. In addition, insulin signaling and cellular stress might be linked with this relationship [29].

### 4.4 Suggestion of future work

For future work, we suggested: (1) Further multi-center follow-up studies to verify the effectiveness of monitoring remnant-C in different populations for future NAFLD risk assessment. (2) Further intervention study to confirm whether reducing remnant-C levels through lifestyle or drugs can reduce the incidence or development of NAFLD or CVD [39]. (3) Further functional research on the underlying mechanism of the relationship between remnant-C and NAFLD. (3) Medical workers or public health decision makers incorporate the joint management of lipid parameters into the disease prevention or treatment programs of NAFLD, and special attentions should be paid to the monitoring role of unconventional lipid parameters, such as remnant-C.

### 4.5 Strengths and limitations

The advantages of this study are as follows: (1) The sample size of this study is large, it has been strictly statistically adjusted, and the final conclusion can be considered to be relatively objective. (2) This is a prospective cohort study which can reflect the causal relationship between remnant-C and NAFLD. (3) Our study first explored the cumulative exposure of remnant-C contributing to NAFLD and performed stratified analyses to assess the effect across various obesity sub-phenotypes. (4) Furthermore, we concluded the nonlinear dose-response effect of remnant-C contributing to NAFLD. A few limitations should also be highlighted: (1) Although ultrasound is the preferred initial non-invasive test for NAFLD, we use ultrasound rather than histology or MRI to diagnose NAFLD which maybe underestimation to some mild hepatic steatosis. (2) The study subjects were the Chinese health check-up population, so the results of this study should be used with caution for other ethnic groups. (3) Serum insulin and C-reaction protein were not measured in this study, so the important role of these in the association could not be clarified. (4) Although the calculation of remnant-C is an economic method that which is especially suitable for chronic disease risk assessment and epidemiological investigation in the general population, the value of remnant-C in our study might have been overestimated by indirect calculation in comparison to direct measurement. More complicated and expensive measurement of remnant-C could be required for accurate results in vulnerable patients. (5) Since the exposure duration is roughly the same, we only focus on the level of remnant-C contributing to NAFLD. In the future, the effect of different exposure duration on the onset of NAFLD will be discussed.

## 5. Conclusion

In conclusion, not only baseline but cumulative exposure of remnant-C was positively associated with NAFLD independent of body shape and metabolic status in the Chinese health check-up population. Remnant-C serves as a promising marker or potential treatment target of NAFLD rather than a traditional risk factor. Therefore, more remnant-C monitoring should be given to individuals for early prevention and intervention in the development and occurrence of NAFLD.

## Supplementary information

**Additional file 1:** **Supplementary Table S1.** Definition and scoring approach Life’s Essential 8 score. **Supplementary Table S2.** Association of baseline remnant-C with risk of NAFLD in Cox proportional hazard models among sub-cohort participants (n = 2,649). **Supplementary Table S3.** Association of baseline remnant-C with risk of NAFLD in subjects without taking lipid-lowering medications (n = 21,757). **Supplementary Table S4.** Association of baseline remnant-C with risk of MAFLD in Cox proportional hazard models (n = 23,766). **Supplementary Table S5.** Association of cumulative exposure to remnant-C with risk of NAFLD (Exam4) in Cox proportional hazard models (n = 2,649). **Supplementary Table S6.** Association of cumulative exposure to remnant-C with risk of NAFLD participants with a minimum of three visits in Cox proportional hazard models (n = 7,718). **Figure S1.** Subgroup analyses of the association of baseline remnant-C with NAFLD.

## Declarations

### Data availability statement

The original contributions presented in this study are included in the article, and further inquiries can be directed to the corresponding author.
