# Supplementary material for: Not only baseline but cumulative exposure of remnant cholesterol predicts the development of nonalcoholic fatty liver disease: a cohort study
Source: Environ Health Prev Med. 2024 Feb 6;29:5. doi: 10.1265/ehpm.23-00289 (PMC10853394; doi:10.1265/ehpm.23-00289)
Supplement: Supplementary file 1 — Additional file 1: Supplementary Table S1. Definition and scoring approach Life’s Essential 8 score. Supplementary Table S2. Association of baseline remnant-C with risk of NAFLD in Cox proportional hazard models among sub-cohort participants (n = 2,649). Supplementary Table S3. Association of baseline remnant-C with risk of NAFLD in subjects without taking lipid-lowering medications (n = 21,757). Supplementary Table S4. Association of baseline remnant-C with risk of MAFLD in Cox proportional hazard models (n = 23,766). Supplementary Table S5. Association of cumulative exposure to remnant-C with risk of NAFLD (Exam4) in Cox proportional hazard models (n = 2,649). Supplementary Table S6. Association of cumulative exposure to remnant-C with risk of NAFLD participants with a minimum of three visits in Cox proportional hazard models (n = 7,718). Figure S1. Subgroup analyses of the association of baseline remnant-C with NAFLD. [file ehpm-29-005-s001.pdf]

Supplemental Material

Title: Not only baseline but cumulative exposure of remnant cholesterol predicts the development of nonalcoholic fatty liver disease: a cohort study

Contents of the Supplementary Appendix

Supplementary Table S1. Definition and scoring approach Life's Essential 8 score.

Supplementary Table S2. Association of baseline remnant-C with risk of NAFLD in Cox proportional hazard models among sub-cohort participants (n = 2,649).

Supplementary Table S3. Association of baseline remnant-C with risk of NAFLD in subjects without taking lipid-lowering medications (n = 21,757).

Supplementary Table S4. Association of baseline remnant-C with risk of MAFLD in Cox proportional hazard models (n = 23,766).

Supplementary Table S5. Association of cumulative exposure to remnant-C with risk of NAFLD (Exam4) in Cox proportional hazard models (n = 2,649).

Supplementary Table S6. Association of cumulative exposure to remnant-C with risk of NAFLD participants with a minimum of three visits in Cox proportional hazard models (n = 7,718).

Figure S1. Subgroup analyses of the association of baseline remnant-C with NAFLD.

Table S1. Definition for Clinical characteristics.

Disease	Definitions
Hypertension[1]	The use of antihypertensive medications, or self-reported physician diagnosis of hypertension, systolic/diastolic blood pressure ≥140/90 mm Hg.
Diabetes[2]	Fasting glucose level ≥7.0 mmol/L, self-reported physician diagnosis of diabetes, or the use of anti-diabetic medication.
Dyslipidemia[3]	The presence of one or more of the following: total cholesterol (TC) ≥ 6.22 mmol/L; low-density lipoprotein cholesterol (LDL-C) ≥ 4.14 mmol/L; density lipoprotein cholesterol (HDL-C) <1.04 mmol/L; triglyceride (TG) ≥ 2.26 mmol/L, or treatment for dyslipidemia.
MAFLD[4]	Fatty liver detected by ultrasonography, in addition to 1 of the following 3 criteria, namely overweight/obesity (body mass index [BMI] ≥ 23 for Asians), presence of type 2 diabetes mellitus (T2DM), or metabolic dysregulation.<br>Metabolic dysregulation is defined as the presence of at least 2 metabolic risk abnormalities:<br>1) waist circumference (WC) greater than or equal to 90/80 cm in Asian men and women;<br>2) blood pressure ≥ 130/85 mm Hg or specific drug treatment;<br>3) plasma triglycerides (TGs) ≥ 1.70 mmol/L or specific drug treatment;<br>4) plasma high-density lipoprotein-cholesterol (HDL-C) < 1.0 mmol/L for men and < 1.3 mmol/L for women or specific drug treatment;<br>5) prediabetes (FBG levels 5.6-6.9 mmol/L).<br>Plasma high-sensitivity C-reactive and homeostasis model assessment of insulin resistance score was unavailable in this study.
Obesity subphenotypes [5, 6]	Participants were classified into 4 obesity subphenotypes according to BMI and metabolic status: Lean or normal weight- metabolically healthy; Lean or normal weight- metabolically unhealthy; Overweight or obesity - metabolically healthy; Overweight or obesity - metabolically unhealthy.<br>1) Lean or normal weight: BMI < 23 kg/m^2;<br>Overweight or obesity: BMI ≥ 23 kg/m^2;<br>2) Metabolically healthy was defined as the presence of one or less metabolic risk abnormalities as listed above; metabolically unhealthy was defined as the presence of at least 2 metabolic risk abnormalities as listed above.

Table S2. Association of baseline remnant-C with risk of NAFLD in Cox proportional hazard models among sub-cohort participants (n = 2,649).

	Baseline Remnant-C quartiles				*P* for trend*
	Quartile 1<br><0.36 mmol/L	Quartile 2<br>0.36–0.47 mmol/L	Quartile 3<br>0.47–0.64 mmol/L	Quartile 4<br>≥ 0.64 mmol/L	
**Total, n**	768	619	561	701	
Case number, n (%)	51 (6.64)	56 (9.05)	82 (14.62)	171 (24.39)	
Incidence rate per 1,000	16.12	22.39	36.77	61.60	
Model 1	1.00 (Reference)	1.24 (0.85–1.82)	1.97 (1.38–2.81)	2.61 (1.88–3.63)	< 0.001
Model 2	1.00 (Reference)	1.12 (0.76–1.65)	1.63 (1.14–2.35)	1.73 (1.20–2.49)	< 0.001
Model 3	1.00 (Reference)	1.15 (0.78–1.69)	1.64 (1.14–2.35)	1.66 (1.15–2.41)	< 0.001

Model 1 was adjusted for age (years), sex.
Model 2 was adjusted for model 1 plus education level, current drinking, current smoking, physical activity, BMI, systolic blood pressure, fasting glucose, triglycerides, estimated GFR, and alanine transaminase at exam1.
Model 3 was adjusted for model 2 plus antidiabetic, lipid-lowering, or antihypertensive medications usage before last exam.
* Test for trend based on variable containing median value for each quarter.

Table S3. Association of baseline remnant-C with risk of NAFLD in subjects without taking lipid-lowering medications (n = 21,757).

	Baseline Remnant-C quartiles				*P* for trend*
	Quartile 1<br><0.35 mmol/L	Quartile 2<br>0.35–0.49 mmol/L	Quartile 3<br>0.49–0.72 mmol/L	Quartile 4<br>≥ 0.71 mmol/L	
Total, n	5397	5649	5296	5415	
Case number, n (%)	393 (7.28)	829 (14.68)	1337 (25.25)	2488 (45.95)	
Incidence rate per 1,000	30.96	61.10	106.82	198.91	
Model 1	1.00 (Reference)	1.64 (1.45–1.85)	2.49 (2.22–2.79)	4.03 (3.61–4.50)	< 0.001
Model 2	1.00 (Reference)	1.49 (1.32–1.68)	2.02 (1.80–2.27)	2.48 (2.20–2.80)	< 0.001
Model 3	1.00 (Reference)	1.50 (1.33–1.69)	2.03 (1.81–2.28)	2.49 (2.21–2.81)	< 0.001

Model 1 was adjusted for age (years), sex.
Model 2 was adjusted for model 1 plus education level, current drinking, current smoking, physical activity, BMI, systolic blood pressure, fasting glucose, triglycerides,

estimated GFR, and alanine transaminase at exam1.
Model 3 was adjusted for model 2 plus antidiabetic, lipid-lowering, or antihypertensive medications usage at exam1.
* Test for trend based on variable containing median value for each quarter.

Table S4. Association of baseline remnant-C with risk of MAFLD in Cox proportional hazard models (n = 23,766).

	Baseline Remnant-C quartiles				*P* for trend*
	Quartile 1<br><0.36 mmol/L	Quartile 2<br>0.36–0.50 mmol/L	Quartile 3<br>0.50–0.74 mmol/L	Quartile 4<br>≥ 0.74 mmol/L	
**Total, n**	6090	5862	5944	5870	
Case number, n (%)	431 (7.08)	900 (15.35)	1512 (25.44)	2774 (47.26)	
Incidence rate per 1,000	32.37	68.77	116.34	219.44	
Model 1	1.00 (Reference)	1.77 (1.458–1.99)	2.63 (2,36–2.93)	4.35 (3.91–4.83)	< 0.001
Model 2	1.00 (Reference)	1.55 (1.37–1.75)	2.11 (1.87–2.37)	2.54 (2.25–2.87)	< 0.001
Model 3	1.00 (Reference)	1.56 (1.38–1.76)	2.12 (1.88–2.38)	2.56 (2.25–2.89)	< 0.001

Model 1 was adjusted for age (years), sex.
Model 2 was adjusted for model 1 plus education level, current drinking, current smoking, physical activity, BMI, systolic blood pressure, fasting glucose, triglycerides, estimated GFR, and alanine transaminase at exam1.
Model 3 was adjusted for model 2 plus antidiabetic, lipid-lowering, or antihypertensive medications usage at exam1.
* Test for trend based on variable containing median value for each quarter.

Table S5. Association of cumulative exposure to remnant-C with risk of NAFLD (Exam4) in Cox proportional hazard models (n = 2,649).

	Cumulative Remnant-C quartiles				*P* for trend*
	Quartile 1<br><0.36 mmol/L	Quartile 2<br>0.36– 0.47 mmol/L	Quartile 3<br>0.47– 0.64 mmol/L	Quartile 4<br>≥ 0.64 mmol/L	
**Total, n**	714	646	648	641	
Case number, n (%)	14 (1.96)	54 (8.36)	62 (9.57)	130 (20.28)	
Incidence rate per 1,000	5.50	23.81	27.44	58.39	

Model 1	1.00 (Reference)	3.96 (2.20–7.15)	3.95 (2.20–7.11)	6.79 (3.85–11.98)	< 0.001
Model 2	1.00 (Reference)	3.78 (2.09–6.84)	3.47 (1.93–6.27)	5.02 (2.77–9.11)	< 0.001
Model 3	1.00 (Reference)	3.79 (2.09–6.86)	3.50 (1.94–6.32)	4.92 (2.71–8.94)	< 0.001

Model 1 was adjusted for age (years), sex.
Model 2 was adjusted for model 1 plus education level, current drinking, current smoking, physical activity, BMI, systolic blood pressure, fasting glucose, triglycerides, estimated GFR, and alanine transaminase at exam1.
Model 3 was adjusted for model 2 plus antidiabetic, lipid-lowering, or antihypertensive medications usage before last exam.
[*] Test for trend based on variable containing median value for each quarter.

Table S6. Association of cumulative exposure to remnant-C with risk of NAFLD participants with a minimum of three visits in Cox proportional hazard models (n = 7,718).

	Cumulative Remnant-C quartiles				*P* for trend*
	Quartile 1<br><0.35 mmol/L	Quartile 2<br>0.35–0.47 mmol/L	Quartile 3<br>0.47–0.66 mmol/L	Quartile 4<br>≥ 0.66 mmol/L	
**Total, n**	2069	1895	1869	1885	
Case number, n (%)	120 (5.8)	215 (11.3)	347 (18.6)	667 (35.4)	
Incidence rate per 1,000	18.18	34.86	57.03	112.47	
Model 1	1.00 (Reference)	1.61 (1.29–2.01)	2.25 (1.83–2.78)	3.76 (3.07–4.60)	< 0.001
Model 2	1.00 (Reference)	1.45 (1.01–2.09)	1.59 (1.11–2.28)	2.02 (1.35–3.02)	< 0.001
Model 3	1.00 (Reference)	1.47 (1.02–2.12)	1.62 (1.12–2.33)	1.98 (1.30–3.00)	< 0.001

Model 1 was adjusted for age (years), sex.
Model 2 was adjusted for model 1 plus education level, current drinking, current smoking, physical activity, BMI, systolic blood pressure, fasting glucose, triglycerides, estimated GFR, and alanine transaminase at exam1.
Model 3 was adjusted for model 2 plus antidiabetic, lipid-lowering, or antihypertensive medications usage before last exam.
[*] Test for trend based on variable containing median value for each quarter.

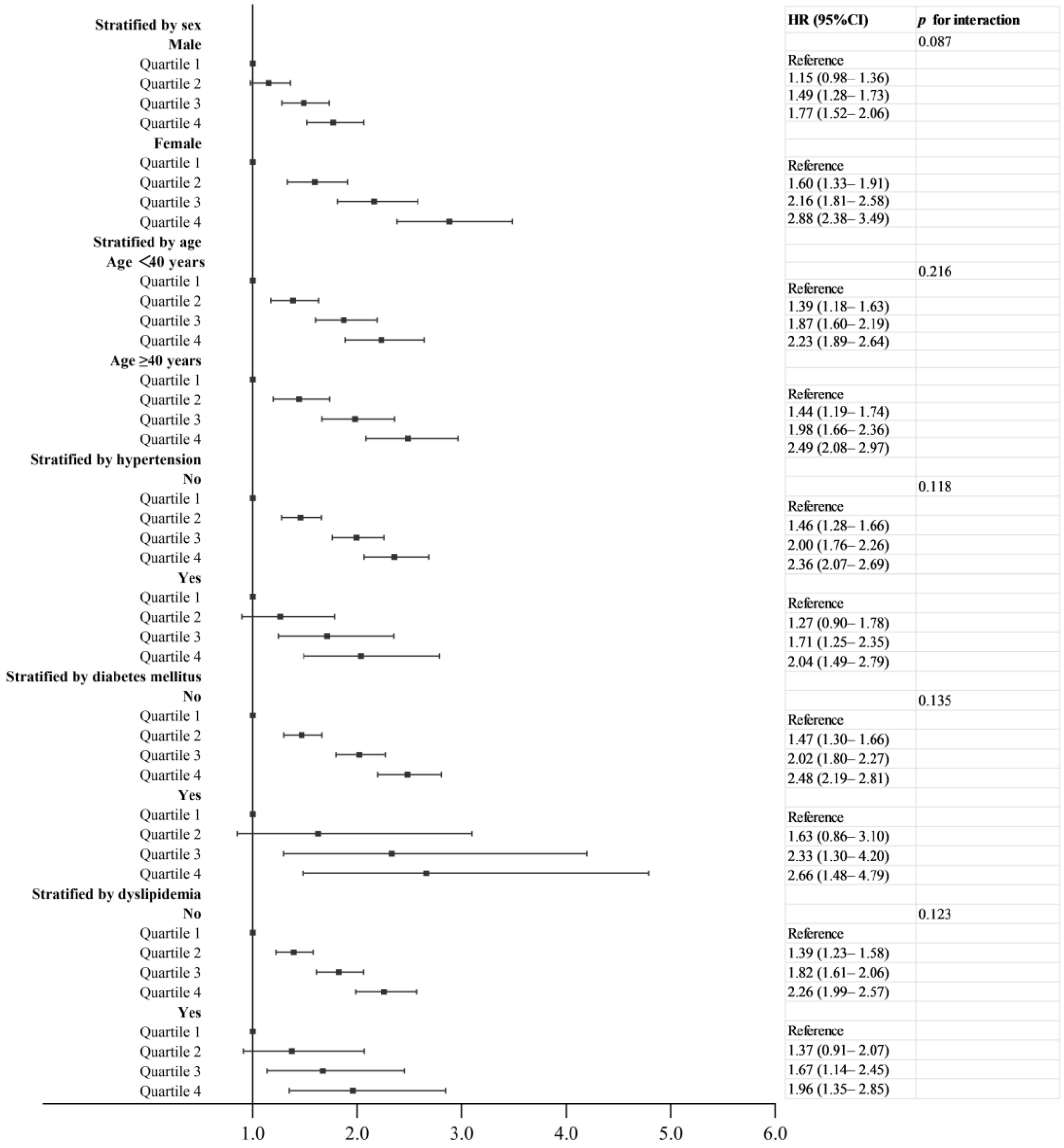

Figure S1. Subgroup analyses of the association of baseline remnant-C with NAFLD. Associations were shown by Cox regression analyses, adjusted variables including age, sex, education level, current drinking, current smoking, physical activity, BMI, systolic blood pressure, fasting glucose, triglycerides, estimated GFR, and alanine transaminase, antidiabetic, lipid-lowering, or antihypertensive medications usage at exam1, in addition to the stratification related factors.

Reference

1. James, P.A., et al., *2014 evidence-based guideline for the management of high blood pressure in adults: report from the panel members appointed to the Eighth Joint National Committee (JNC 8).* JAMA, 2014. **311**(5): p. 507-20.
2. Kerner, W., J. Bruckel, and A. German Diabetes, *Definition, classification and diagnosis of diabetes mellitus.* Exp Clin Endocrinol Diabetes, 2014. **122**(7): p. 384-6.
3. Yang, W., et al., *Serum lipids and lipoproteins in Chinese men and women.* Circulation, 2012. **125**(18): p. 2212-21.
4. Eslam, M., et al., *A new definition for metabolic dysfunction-associated fatty liver disease: An international expert consensus statement.* J Hepatol, 2020. **73**(1): p. 202-209.
5. Rui, X. and Jiangao, F., *Brief introduction of an international expert consensus statement:A new definition of metabolic associated fatty liver disease.* Journal of Clinical Hepatology (China), 2020. **36**(6): p. 1224-1227.
6. Ye, Q., et al., *Global prevalence, incidence, and outcomes of non-obese or lean non-alcoholic fatty liver disease: a systematic review and meta-analysis.* Lancet Gastroenterol Hepatol, 2020. **5**(8): p. 739-752.
